# Annotation of a hybrid partial genome of the coffee rust (*Hemileia vastatrix*) contributes to the gene repertoire catalog of the Pucciniales

**DOI:** 10.3389/fpls.2014.00594

**Published:** 2014-10-31

**Authors:** Marco A. Cristancho, David Octavio Botero-Rozo, William Giraldo, Javier Tabima, Diego Mauricio Riaño-Pachón, Carolina Escobar, Yomara Rozo, Luis F. Rivera, Andrés Durán, Silvia Restrepo, Tamar Eilam, Yehoshua Anikster, Alvaro L. Gaitán

**Affiliations:** ^1^Plant Pathology, National Center for Coffee Research – CENICAFÉChinchiná, Colombia; ^2^Departamento de Ciencias Biológicas, Universidad de los AndesBogotá, Colombia; ^3^Institute for Cereal Crops Improvement, Tel Aviv UniversityTel Aviv, Israel

**Keywords:** genome, coffee rust, coffee, plant pathogens diversity, RNA-seq, genetic variants

## Abstract

Coffee leaf rust caused by the fungus *Hemileia vastatrix* is the most damaging disease to coffee worldwide. The pathogen has recently appeared in multiple outbreaks in coffee producing countries resulting in significant yield losses and increases in costs related to its control. New races/isolates are constantly emerging as evidenced by the presence of the fungus in plants that were previously resistant. Genomic studies are opening new avenues for the study of the evolution of pathogens, the detailed description of plant-pathogen interactions and the development of molecular techniques for the identification of individual isolates. For this purpose we sequenced 8 different *H. vastatrix* isolates using NGS technologies and gathered partial genome assemblies due to the large repetitive content in the coffee rust hybrid genome; 74.4% of the assembled contigs harbor repetitive sequences. A hybrid assembly of 333 Mb was built based on the 8 isolates; this assembly was used for subsequent analyses. Analysis of the conserved gene space showed that the hybrid *H. vastatrix* genome, though highly fragmented, had a satisfactory level of completion with 91.94% of core protein-coding orthologous genes present. RNA-Seq from urediniospores was used to guide the de novo annotation of the *H. vastatrix* gene complement. In total, 14,445 genes organized in 3921 families were uncovered; a considerable proportion of the predicted proteins (73.8%) were homologous to other Pucciniales species genomes. Several gene families related to the fungal lifestyle were identified, particularly 483 predicted secreted proteins that represent candidate effector genes and will provide interesting hints to decipher virulence in the coffee rust fungus. The genome sequence of Hva will serve as a template to understand the molecular mechanisms used by this fungus to attack the coffee plant, to study the diversity of this species and for the development of molecular markers to distinguish races/isolates.

## Introduction

Coffee rust caused by the fungus *Hemileia vastatrix* (Hva) leads to widespread damage to crops worldwide. The disease develops polycyclic epidemics in a season, which means there is an overlapping succession of infection cycles. Under tropical conditions and in semi-perennial plants, such as coffee, Hva poses a permanent threat for producers. In the absence of control methods, the pathogen has been reported to cause losses of up to 30% in susceptible varieties of the species *Coffea arabica* during mild epidemics (Monaco, 1977; Rivillas et al., 2011). The fungus, which is a biotroph that targets *Coffea* as the single known host genus, spread from Africa and was responsible for the collapse of coffee production in India and Ceylon by the mid XIX century. It arrived in America in 1970, and since then, it has quickly disseminated to all the other coffee-producing areas of the continent. In Colombia, Hva was reported for the first time in 1983 in the central coffee-producing zone of the country (Leguizamón et al., 1984). The presence of Hva in almost every coffee plantation in the world has been one of the main drivers for plant breeders to release rust-resistant varieties. Recent outbreaks of the disease have affected major areas of coffee production in Colombia (Rozo et al., 2012) and Central America (Cressey, 2013), and the evidence linked these new epidemics to changes in weather patterns, including rainfall distribution and quantity (Cristancho et al., 2012).

Multiple Hva races have been reported throughout the world (Rodrigues et al., 1975; Carvalho et al., 1987), and studies from the CIFC (Coffee Rust Research Center) in Portugal have identified over 30 races of the pathogen using a series of more than 40 differential coffee genotypes (Rodrigues et al., 1993). Historically, race II has been predominant in most countries, and it attacks all cultivated varieties of the species *C. arabica* that have not been bred for disease resistance (Rodrigues et al., 1975). In addition to race II, 6 other physiological races have been identified in Colombia using a set of differential plants developed at CIFC (Oeiras, Portugal), attacking some lines of the resistant cultivars (Castillo and Leguizamón, 1992; Gil and Ocampo, 1998; Alvarado and Moreno, 2005; Rozo et al., 2012). At least 10 more isolates not differentiated by CIFC differential plants, remain to be characterized in Colombia, and several other unknown isolates have also been detected elsewhere (Gouveia et al., 2005).

The emergence of new races and more aggressive isolates in plant pathogens threaten agriculture worldwide as recently observed with the wheat stem rust fungus and the new epidemics of coffee rust, which clearly indicate that further detailed studies and continuous monitoring are needed to improve integrated disease management strategies that mitigate their destructive effect (Aime et al., 2006). Being obligate pathogens, the study of rust fungi biology is particularly challenging and needs substantial investments given the fact that a large set of differential plants have to be employed for the classification of races. The development of novel tools for the identification of isolates is critical to study the biology of these major plant pathogens and genomics might offer such tools. Differential Hva genes expressed in urediniospore, appressoria, and haustoria have already been identified (Fernandez et al., 2012; Talhinhas et al., 2014).

Genomic studies of plant pathogens have provided insights into their evolution, the mechanisms that generate genetic variability and the repertoire of genes that are involved in pathogenesis. These studies have shown that rust fungi exhibit very large genome sizes [*Melampsora lini* = Mli (Nemri et al., 2014) is the largest fungal genome so far] compared to other fungi, containing very large numbers of genes, over 16,000 in most cases, compared to other fungi groups such as Ustilaginomycotina [*U. maydis* = 6786 protein coding genes (Schirawski et al., 2010)] or other non-rust Pucciniomycotina such as *Mixia osmundae* (Toome et al., 2014). Rusts also show a large content in transposable elements (i.e., nearly 50%, in the genomes analyzed so far, Duplessis et al., 2014). All these features indicate that rust genomes in general are complex to sequence and assemble due to the repetitive content and large genome size.

The discovery of predicted secreted virulence determinants in plant pathogens has also been possible through genomic analysis. Secreted proteins have been linked to the virulence of plant-pathogenic fungi (Spanu, 2012) and many have been predicted in *Melampsora* spp. (Joly et al., 2010; Hacquard et al., 2012), *P. striiformis* (Cantu et al., 2011, 2013), *P. graminis* (Duplessis et al., 2011), and *H. vastatrix* (Fernandez et al., 2012). Thus, the discovery of the secreted protein genes (Saunders et al., 2012) and the functional demonstration of their decisive role in the infection process help in unraveling previously unknown mechanisms of pathogenicity that operate in biotrophic fungi.

We have obtained genome and transcriptome sequences of the coffee rust fungus, and we expect these data to allow the identification of potential molecular markers for the study of rust isolates/races. The knowledge of the Hva genome and particularly of its secretome is a critical point for understanding the mechanisms used by the fungus during the colonization of coffee tissues and allows for comparisons of pathogenesis processes in other rust fungus-plant interactions. The chimeric genome assembly obtained was further used to define polymorphism between isolates and to analyse its basic contents such as the gene complement and TE families. The predicted proteome was additionally supported by a transcriptome analysis of Hva urediniospores. Within the predicted gene complement, a more precise analysis was performed on predicted secreted proteins, likely containing Hva candidate effectors.

## Results

### Nuclear DNA content

The nuclear DNA content of 10 Hva samples was measured by Flow cytometry analysis (Table 1). Two groups could be distinguished among the 10 samples: one contains four samples that showed a lower content of 1.17–1.29 pg of DNA and a second with the six remaining samples showed a higher content of 1.55–1.76 pg of DNA per urediniospore (~30% more). Based on these results and compared to the genome size of *P. triticina* used as a control (135 Mb, Puccinia Group Genomes Database, Broad Institute), we estimate the genome size of Hva to be 243–324 Mb.

**Table 1:** **Nuclear DNA content of Hva urediniospore samples measured by FCM**.

***Coffea* species and genotypes**	**Geographical location**	**DNA Content (pg)**	
		**Group 1**	**Group 2**
*C. arabica* var. Caturra	La Alcancía, Antioquia	–	1.63
*C. arabica* var. Caturra	El Cedral, Pereira, Risaralda	–	1.55
*C. arabica* var. Caturra	Santa María, Antioquia	1.29	–
*C. arabica* var. Caturra (Acc. 1421)	Chinchiná, Caldas	1.17	–
*C. arabica* BA-13	Chinchiná, Caldas	–	1.76
*C. arabica × C. canephora*–-Timor Hybrid H-419/2	Chinchiná, Caldas	–	1.62
*C. arabica × C. canephora*–-Timor Hybrid H-584	Chinchiná, Caldas	1.21	–
*C. arabica* var. Tipica	Chinchiná, Caldas	1.18	–
*C. arabica* var. Mundo Novo	Chinchiná, Caldas	–	1.70
*C. liberica*	Chinchiná, Caldas	–	1.70
	Mean	1.21	1.66
	Standard deviation	0.05	0.08
	CV%	5.87	6.41

*Hva urediniospore samples were stained with Propidium Iodide for Flow Cytometry fluorescence measures following the protocol described by Eilam et al. (1994)*.

### Genome assemblies

We sequenced the genomes of the following isolates: HvCat, Hv387, Hv949, HvDQ952, HvH179, HvH569, HvH701, and HvMar; for isolate HvCat, Illumina and 454 sequencing were combined. We obtained a total of 412 million short-reads from Illumina and 5.8 million reads from 454. The fraction of reads that passed quality filtering was over 85% in all Hva Illumina sequenced samples but only 52% for the 454 Hva sequenced sample (Table S1, see methods section for filtering parameters). A hybrid 454-Illumina assembly was obtained, combining all genomic Hva sequences with the script clc_novo_assembly from the assembler suite CLC Assembly Cell v4.0.1 (CLC bio, Aarhus, Denmark); we have also performed separate assemblies of the genomes of each isolate. Unfortunately due to inherent characteristics and composition of the genomes (low GC content and richness in TE, detailed in the following sections), we were only able to obtain partial genome assemblies. In order to improve the overall genome assembly, our strategy was to generate an hybrid assembly that takes into account all reads obtained from the 8 isolates together to produce a unique sequence that is a chimera of the sequenced isolates. We were able to define a genome sequence of 333 Mb (129X sequencing depth) composed of 396,264 contigs and 302,466 scaffolds.

We assessed the completeness of the hybrid and individual genomes by running CEGMA with a set of 248 ultra-conserved Core Eukaryotic Genes (CEGs) (Parra et al., 2007) (Table 2). Statistics for the hybrid assembly are shown in Table 3; based on the Hva chimeric assembly data, and using Jellyfish (Marçais and Kingsford, 2011) to compute the number of distinct k-mers of different lengths and their relationship to coverage, we estimated the size of the Hva genome to be 333 Mb. Considering that the HvaHybrid genome sequence was the only one with a fairly good coverage of conserved core genes, we decided to use it as the reference genome for further analysis.

**Table 2:** **Statistics gathered from the genome assemblies of Hva individual isolates and the hybrid assembly**.

**Sample IDs**	***Coffea* species and genotypes**	**Raw reads**	**Clean reads^a^**	**Contigs**	**Assembly size**	**Completeness^b^**
HvHybrid^c^		412,417,464	359,076,496	396,264	333,258,024	91.94%
HvCat 454^d^		5,860,446				
HvCat Illumina	*C. arabica* var. Caturra	48,396,016	43,704,716	254,645	122,820,521	57.26%
Hv387	*C. canephora* CII56	58,593,986	50,782,526	211,495	150,707,107	44.35%
Hv494	H89: *C. arabica* var. Bourbon resistant × *C. arabica* CaRCV3	55,326,774	47,738,686	211,728	138,293,025	35.08%
HvDQ952	F2 – *C. arabica* var. Caturra × HdT 1343 (*C. arabica* × *C. canephora*)	43,875,056	38,844,164	197,927	121,119,448	31.85%
HvH_179	H3101: (*C. arabica* CaCV1 × Hdt (*C. arabica* × *C. canephora*) 1343 574CV2) × *C. arabica* CtyR	49,025,718	42,033,780	203,770	131,574,289	31.05%
HvH_569	H3041: (*C. arabica* × HarrarR3) × HdT(*C. arabica* × *C. canephora*) 1343 Africa 1386	51,960,392	45,000,606	202,168	133,358,100	37.50%
HvH_701	H2094: (*C. arabica* MundoNovo) × F502 (*C. arabica* accession from Tanganica)	60,634,018	53,080,264	215,628	158,292,515	56.86%
HvMar_1	*C. arabica* var. Caturra	44,605,504	39,408,690	203,360	125,814,765	22.18%

a *Clean reads were obtained after quality trimming and removal of duplicates*.

b *Completeness of the genome was calculated running the software CEGMA with a set of 248 ultra-conserved Core Eukaryotic Genes (CEGs) (Parra et al., 2007)*.

c *The hybrid assembly was generated by the combination of all short reads from the eight isolates*.

d *Eight and a half plates were sequenced with 454 technology*.

**Table 3:** **Summary of the Hva genome hybrid assembly**.

N° Contigs assembled		396,264
N° Scaffolds assembled		302,466
Total residues assembled		333,481,311
Length	Max	85,126
	Average	841.56
	N50	1,59
Reads	Total	336,649,188
	Unassembled	197,88,611
	Assembled	316,860,577
	Multihit	37,520,793
	Potential pairs	
	Paired	78,105,740
	Not Paired	255,469,308

*The 454 and Illumina clean reads were assembled and the same reads were mapped against this set of assembled contigs using the software CLC Assembly Cell v4.0.1*.

**Figure 1:**
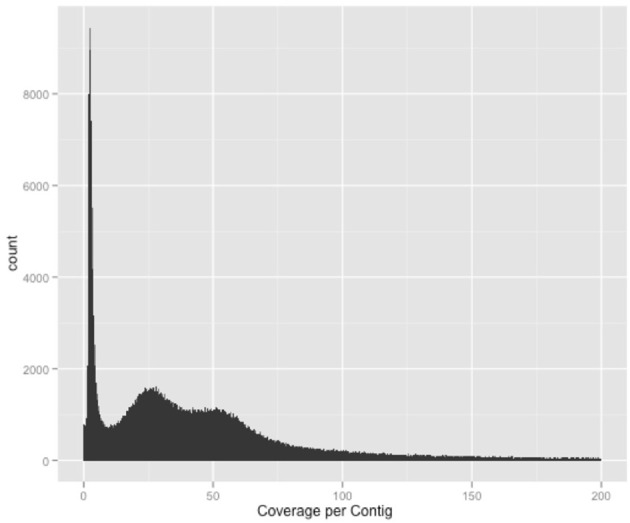
**Plot representing the reads coverage per contig in the HvHybrid assembly**. Low read coverage was identified in most contigs.

**Figure 2:**
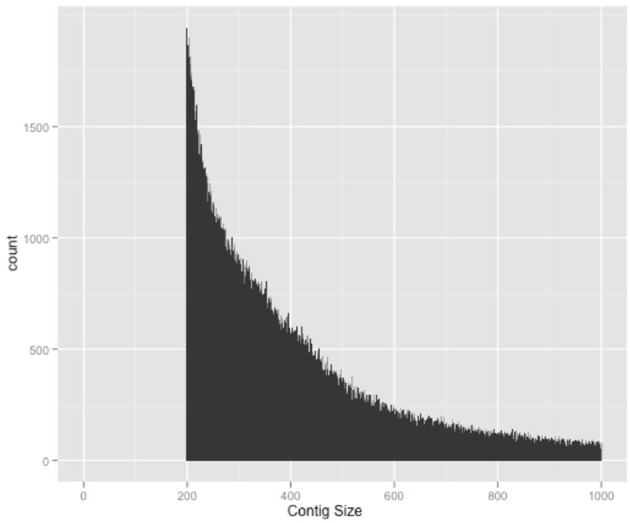
**Association between number of reads and size of contigs in the HvHybrid Assembly**. Over-coverage was identified in short contigs.

**Figure 3:**
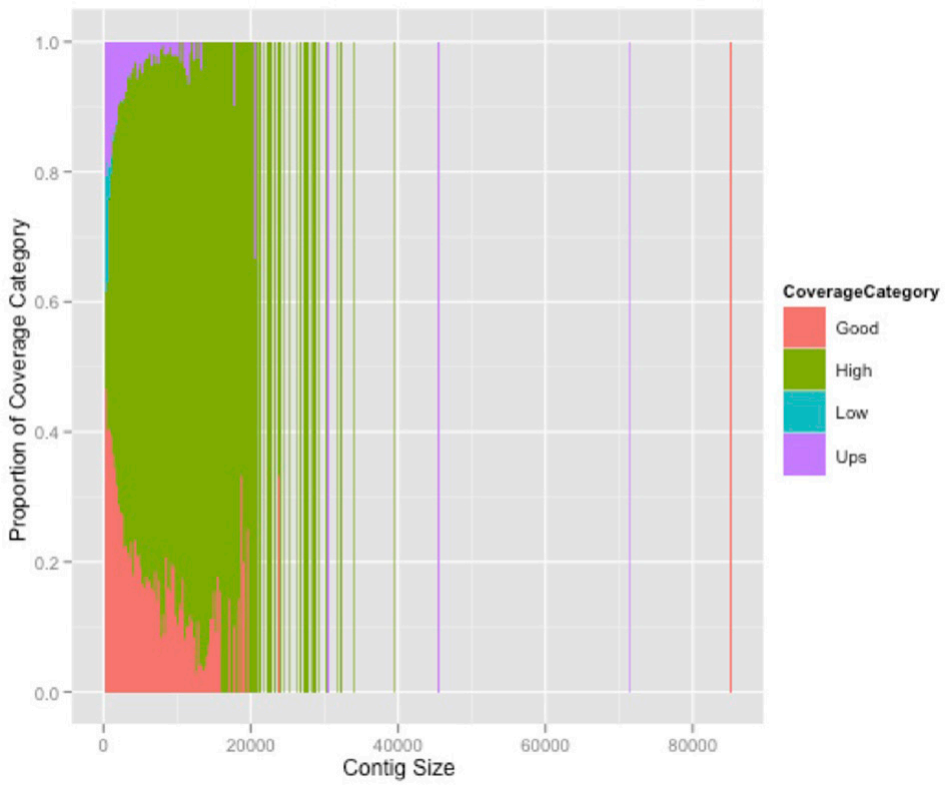
**Association between contig size and coverage**. Contigs were clustered by color by running an R script: 0–5 low coverage, 5–45 good coverage, 45–100 high coverage and more than 100 over-coverage.

**Figure 4:**
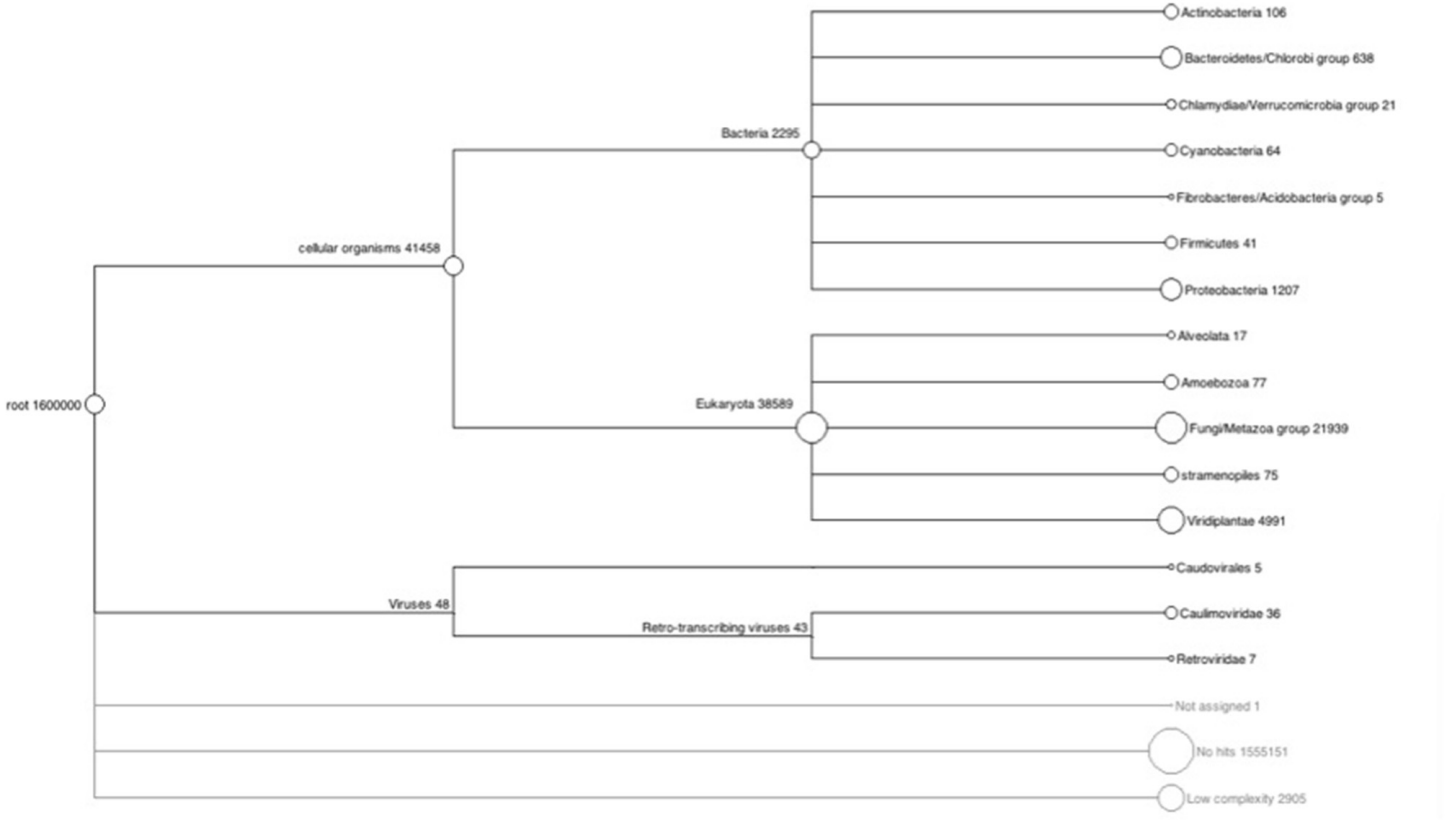
**Analysis of sequence contamination in the HvHybrid assembly**. The HvHybrid assembled contigs were searched for contaminants with the program Megan4 (Huson et al., 2001). Each node is labeled by a taxon and the number of reads assigned to the taxon. The size of a node (circle size) is scaled logarithmically to represent the number of assigned reads. A low bacterial and plant sequence contamination was identified in the assembly.

A total of 23.2% of the paired-end reads mapped in the same contig of the HvaHybrid genome assembled. Most unpaired reads (66.8%) matched two different contigs (useful for scaffolding, data not shown). Several contigs displayed coverage greater than 100X, but most of the contigs exhibited low coverage (Figure 1); however, over-coverage was pronounced in the short contigs (Figure 2). We also illustrated the range of coverage of the contigs and its association to the contigs size (Figure 3). The largest contig, Hvcontig_23458 (85 Kbp) showed good coverage. However, the next two contigs in size, Hvcontig_171 (45 kbp) and Hvcontig_161 (71 kbp), showed over-coverage and belong to the Hva mitochondria (see below). Bacterial contamination was very low representing less than 1% of the sequences (Figure 4).

### Mitochondrial genome annotation

**Figure 5:**
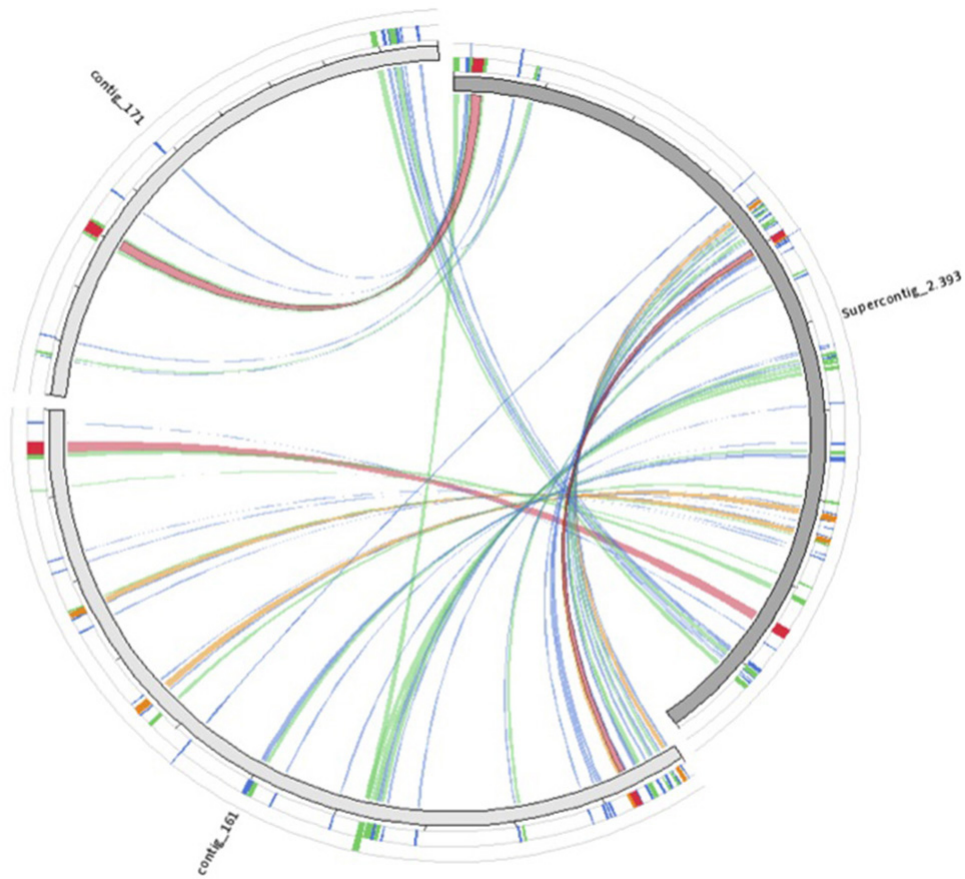
**Homology of Hva mitochondrial sequences to the mitochondrial genome of PGT**. Clone_161 and clone_171 sequences from HvHybryd assembly were compared to the mitochondrial genome of *P. graminis* f.sp *tritici* (Supercontig_2.393; 79,2Kb) by running BLAST sequence similarities (*e* = 1e-5). Homologous regions are illustrated with Circoletto (Darzentas, 2010).

We used the *P. graminis* f.sp. *tritici* (PGT) (Puccinia Group Genomes Database, Broad Institute) mitochondrial genome to find homologs in the *HvHybrid* assembly by BLAST (*e* = 1e-5). BLAST sequence similarities were pictured using the visualizing tool Circoletto (Darzentas, 2010). As shown in Figure 5, Hvcontig_161 (71,379 bp, %GC = 33%) and Hvcontig_171 (45,581 bp, %GC = 35%) cover over 70% of the mitochondrial genome of PGT. We also found that 7 contigs of the HvCat assembly entirely covered the mitochondrial genomes of PGT (79,748 bp) and the soybean rust *Phakopsora pachyrhizi* (31,825 bp; Stone et al., 2010) (results not shown). From this analysis we estimate that the Hva mitochondrial genome it is at least of the size of the PGT mitochondrial genome.

### De novo identification of transposons

The genomes of rust fungi sequenced to date all contain large numbers of transposable elements, which is a major problem for proper assembly (Duplessis et al., 2011; Zheng et al., 2013). A careful annotation of TEs was performed in the chimeric assembly and it showed that the Hva genome also contained a large proportion of repeated sequences. Interspersed repeats were identified in 74.4% of the Hva assembled hybrid contigs using the algorithm RepeatMasker. A similar fraction of repeats was identified in the individual assemblies (71–74%). A large proportion of repeats were classified as LTR elements (38.7%), a smaller proportion was classified as DNA elements (7.2%), only 2.1% were classified as LINEs, and 26.3% of repeats were unclassified.

The sequences were annotated for the presence of LTRs, direct repeats and inverted repeats as well as sequence similarities to repeat sequences from Pucciniales. Four novel retrotransposon families were identified in the Hva genome (Table S2).

### Predicting protein-coding genes

In order to capture the gene space of the coffee rust genome, we performed a transcriptome analysis of freshly harvested urediniospores based on Illumina RNA-Seq. A total of 44,297–64,752 transcripts could be identified in the three RNA-seq based libraries with the program Trinity, with the HvCatNor normalized library holding the smallest number of genes (Table S3). We aligned those transcripts onto the chimeric and individual samples assemblies and showed that the normalized library contains the largest fraction of Hva expressed mapped genes (Table S4). The normalization approach decreases the prevalence of high abundance transcripts and equalizes transcript concentrations in a cDNA sample, thereby increasing the discovery of low abundance transcripts. The level of contamination of the Hva RNA-seq datasets with plant, bacterial and other contaminant sequences was moderate; the fraction of contamination for each sample was 13.6% for the normalized library HvCatNor, 12,3% for the HvH420_701 library, and 18,9% for the HvCat955 library (Table S5). We carried out homology annotation with BLAST against Hva germinating urediniospore transcripts dataset and other rusts predicted proteins datasets (Table 4). Our Hva urediniospores dataset is as expected very similar to the Hva germinating urediniospores transcripts identified by Talhinhas et al. (2014).

**Table 4:** **Homology of Hva transcript datasets to Pucciniales predicted proteins**.

		**Hva urediniospore transcripts**		
**Hva samples^a^**	**Assmbled transcripts**	**HvCatNor**	**HvH420_701**	**HvCat955**
**I**				
Hva (gU)	4267	91.0% (3884)	93.1% (3973)	93.9% (4007)
*H. vastatrix* (Ap)	3627	59.2% (2147)	63.1% (2290)	63.8% (2315)
*H. vastatrix* (H)	4465	50.0% (2232)	49.1% (2202)	49.6% (2229)
**Rust species**	**Predicted proteins**			
**II**				
*Pt*	11,630	42.2% (18,703)	40.0% (22,240)	36.6% (23,705)
*Pgt*	15,979	43.8% (19,411)	41.5% (23,129)	37.8% (24,480)
*Pst*	22,815	43.5% (19,257)	40.4% (22,544)	36.9% (23,874)
*Mlp*	16,694	39.2% (17,385)	40.8% (22,765)	37.8% (21,302)

*I. Homology sequence analysis between Hva transcriptomes datasets (this study) and Hva germinating urediniospores, appresoria, and haustoria transcript sequences (gU, Ap, H, Talhinhas et al., 2014) was performed with the program BLASTn and an E = 1e^−20^. II. BLASTx sequence similarity analysis of H. vastatrix RNA-seq sequences and other rust predicted protein datasets (E = 1e^−3^). Numbers represent fraction (%) of hits found*.

a *Hva samples described in Fernandez et al. (2012)*.

The predictions of the gene coding space was performed using TopHat (Trapnell et al., 2009) for mapping of RNA-seq data against the HvHybrid genome assembly and proteins were predicted with Augustus (Stanke and Waack, 2003; size filter = 70 amino-acids), using the RNA-Seq data as a guide. We identified a total of 21,345 contigs that matched the RNA-seqs and we predicted a total of 18,234 protein sequences with an average length of 1047 bp for the gene models. We identified 13,796 Hva protein homologs (73.86%) in the Pucciniales order (67,118 sequences) using blastp (*e* = 1e-3).

The total set of sequences was filtered to remove repeats identified in RepBase Release 17.01, and this resulted in a final set containing 14,445 predicted protein-coding gene sequences. Over 96% of this set of gene models was identified in the individual assemblies (Table S6). We explored this set of predicted proteins searching for KOGs in the NCBI Conserved Domain Database; 8458 Hva protein-coding genes having a KOG homolog were functionally annotated (Table S7). A total of 3921 gene families with 2–66 gene members were identified with OrthoMCL (Table S8); we also identified 2103 orphan genes.

### Secretome annotation

**Figure 6:**
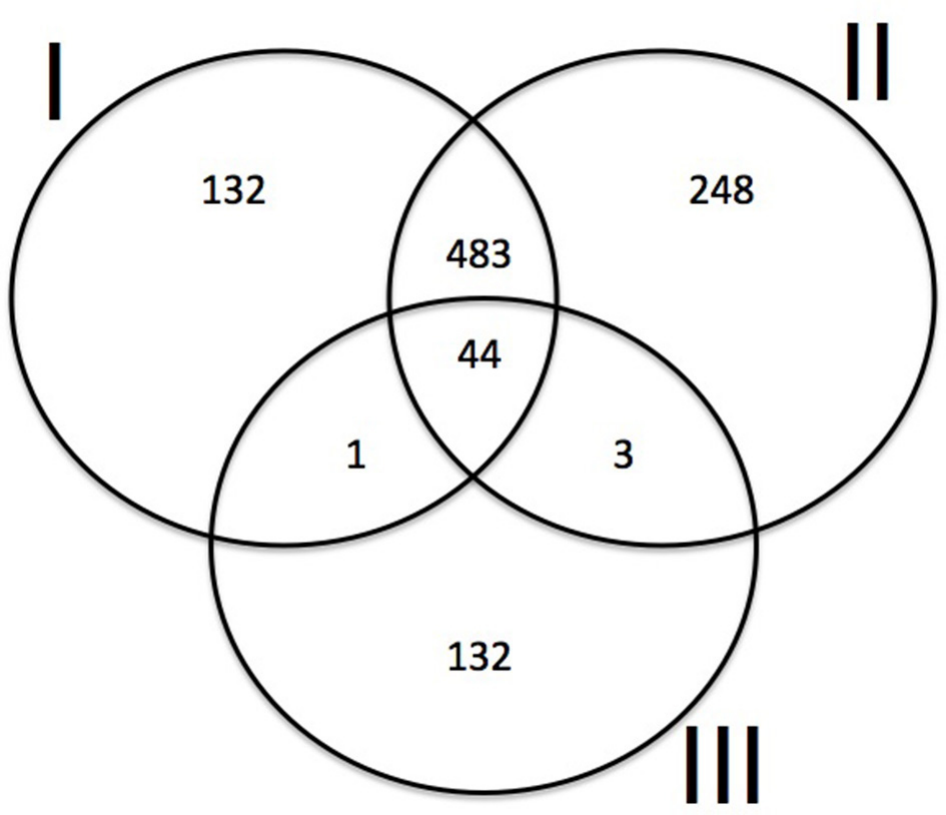
**Venn diagram showing the consensus set of Hva secreted predicted proteins**. PProwler (0.9 probability cut-off) and SignalP were used to predict secreted proteins. A set of 14,445 putative proteins was used for classification into secreted and non-secreted proteins. The results were compared with Hva secreted proteins predicted by Fernandez et al. (2012). Hva secreted proteins predictions: **(I)** PProwler (this study). **(II)** SignalP (this study). **(III)** Secreted proteins predicted by Fernandez et al. (2012).

We predicted 659 secreted proteins using PProwler and 775 secreted proteins with the SignalP algorithm. A total of 180 proteins in our Hva set presented homologs with the secreted proteins already predicted in Hva (Fernandez et al., 2012). A Venn diagram (Figure 6) showed shared and unique coincidences between the three sets of data, including 44 proteins extracted by comparison with the dataset predicted by Fernandez et al. (2012). Most of the secreted proteins predicted are organized in gene families and they were mapped with tblastx to at least one contig from each of the individual assemblies; a final set of 28 predicted proteins was obtained after filtering those belonging to the same gene family and they were functionally annotated with blastp against swissprot, RefSeq, Uniref100 and the non-redundant protein sequences databases (Table S9). Only five sequences did not have a homolog sequence with other Pucciniales fungi. Six sequences had a homolog already identified as a secreted protein in *M. larici-populina*. We did not identify in the genome of Hva a homolog of ps87 of *P. striiformis* f.sp. *tritici*, a conserved secreted protein in several fungal plant pathogens (Gu et al., 2011). However, an identical copy of the Hva RTP1 gene (GenBank: FR851895), a transferred protein belonging to the family of effectors in rusts (Pretsch et al., 2013) was identified, suggesting that some effectors are very well conserved between different rust species (Spanu, 2012).

We identified homologs of the predicted secreted proteins in all but one of the Hva individual assemblies; a homolog of protein KF018005 was not identified in the assembly of HvCat. The secreted proteins mapping to the individual assemblies showed that 6 proteins were identical in every Hva sample. The remaining 22 predicted proteins displayed polymorphism in at least two isolates (Table S10).

For Hva proteins KF018008, KF018015, KF018016, KF018020, KF018028 we did not find evidence of predicted homologs in other rusts, representing unique genes of the coffee-rust interaction not represented in other pathosystems. Interestingly, protein KF018020 had no homolog in any database and we could not detect polymorphisms of this protein-coding gene in the Hva individual assemblies, but the Hva genome holds 32 copies of this gene. Protein KF018028, represented by a single copy in the genome, is surprisingly the most diverse of the Hva secreted proteins. It is worth noting that it does not have homologs in any other organism.

### Protein kinases (PKs)

Given the fact that PKs are involved in essential pathways related to development and adaptation to different environments (Miranda and Barton, 2007), we determined differences between PKs families present in pathogen and non-pathogen basidiomycetes. A total of 210 PKs were identified within the set of Hva predicted proteins using HMM3 (Eddy, 2011).

This set of sequences was compared against the predicted PKs of other Pucciniales showing that most protein kinases, including gene families coding for signal transduction pathways, are shared between rust genomes (Table S11). We wanted to know the protein kinases exclusively found in pathogenic basidiomycetes and for that we run a BLASTp homology search of PKs of five pathogenic species, *H. vastatrix, P. graminis* f.sp *tritici*, *P. triticina*, *M. larici-populina*, and *U. maydis* against predicted PKs of the non-pathogenic basidiomycetes *Coprinopsis cinerea* (Stajich et al., 2010) and *Laccaria bicolor* (Martin et al., 2008). There are 18 PKs unique to the genomes of plant pathogenic fungi (Table S12) and we identified 236 PKs sequences present in *C. cinerea* and *L. bicolor* but not in pathogenic fungi. In the later group we highlight functions TKL/TKL, PKL/ccin9, PKL/CAK/Fmp29, FunK1, AgaK1, atypical/PIKK/TRRAP, and atypical/HisK PKs families because they were not identified in any of the pathogen species although they are expanded in unique families in *C. cinerea* (Stajich et al., 2010) and *L. bicolor* (Martin et al., 2008).

## Discussion

We have generated a de novo hybrid genome assembly of the coffee rust from the sequence of 8 rust samples. We also assembled transcript sequences obtained from normalized and non-normalized RNA-seq libraries representing the urediniospore stage of the fungus. The hybrid genome assembly was the most comprehensive in terms of capturing the largest proportion of the gene space in Hva, therefore offering a picture of a chimeric genome of this species. There appears to be a large extent of repetitive sequences in this chimeric genome, which was evident in our hybrid assembly, as shown by over-covered contigs. For example, two of the three largest contigs showed over-coverage and this event was also found in most contigs shorter than 400 bp. Contamination analysis showed the presence of Bacteria and Viridiplantae sequences in the sequencing reads but the fraction of these sequences was very low. The limitation of having to collect spore samples from plant tissue renders it impossible to have samples free of other organisms; consequently, finding bacterial, plant and other DNA sequences was not unexpected.

We estimated the Hva genome size to be 243–324 Mb by FCM and the assembled scaffolds size was close to the larger figure (333 Mb). Differences between the relative amounts of DNA measured by FCM might be due to the presence of a mixture of different cells containing different number of nuclei in the Hva samples tested; Hva urediniospores in coffee leaves carry out meiosis giving rise to spores at different stages of development containing unequal numbers of nuclei in a process referred as cryptosexuality (Carvalho et al., 2011). A similar mechanism of parasexual recombination has been described in *P. triticina* (Wang and McCallum, 2009). The haplophase has not been recognized in Hva and only urediniospores and teliospores representing the dikaryophase have been identified (De Castro et al., 2009).

Our Hva genome size estimates fall remarkably short of recent measurements of 733 Mbp (Carvalho et al., 2014) and 796.8 Mbp (Tavares et al., 2014) obtained by FCM of Hva nuclei isolated from urediniospores. Highly repetitive genomes such as the Hva genome are complex to sequence and analyze and genomes with a high content of repeats are difficult to sequence completely (Sun et al., 2003). Overall, the Hva genome size is larger compared with other fungal genomes, including the Basidiomycetes *M. larici-populina* (101.1 Mb), *P. graminis* f.sp. *tritici* (88.6 Mb) (Duplessis et al., 2011), and *Laccaria bicolor* (68.9 Mb) (Martin et al., 2008), the arbuscular mycorrhizal fungus *Rhizophagus irregularis* (153 Mb) (Tisserant et al., 2013), and the Ascomicota *Tuber melanosporum* (125 Mb) (Martin et al., 2010), and *Blumeria graminis* f.sp. *tritici* (174 Mb) (Parlange et al., 2011).

Our analysis indicated that the Hva mitochondrial genome is at least the size of the *P. graminis* mitochondria. The different genome size estimates obtained so far make imperative the assembly of an Hva genome from a single Hva isolate to clearly elucidate the real nuclear genome size, mitochondrial genome size and fraction of repetitive sequences for this fungus.

We identified a large fraction of repetitive sequences in the hybrid genome; 74.4% of the assembled contigs contain repetitive sequences, with most of them representing transposable elements. Because of the hybrid nature of the assembly, this might be and over-estimate of the real fraction of repetitive sequences present in the genome. However, given the large estimates for the Hva genome size, we expect the genome sequence to contain a large proportion of repeats. A high proportion of transposable elements have also been identified in the genomes of other rusts (Duplessis et al., 2011; Zheng et al., 2013) and the plant pathogen *Blumeria graminis* (Spanu et al., 2010). Genome expansion caused by the replication of TEs has been shown to occur in filamentous fungal and oomycete pathogens of plants, and some expansion of virulence-related genes are associated with their large genome size (Kemen and Jones, 2012). The high diversity of many *Avr*-genes in the rice blast fungus *Magnaporthe grisea* is related to their association with repeated sequences (Huang et al., 2014). On the other hand, the non-pathogen basidiomycetes *L. bicolor* (Martin et al., 2008) and *C. cinerea* (Stajich et al., 2010) harbor a much-reduced proportion of repeated sequences. Whether the genome of Hva has suffered an expansion of virulence-related genes mediated by transposition events should be investigated in further detail. Given the fact that a hybrid genome might contain an over-representation of the fraction of repetitive elements present in single genomes, there is still need to be cautious about the final proportion of repeats in the Hva genome.

The assembly exhibited a high level of fragmentation as shown by the large number of scaffolds obtained in the final assembly and the low N50 value. This fragmentation can be explained by the highly repetitive nature of the Hva genome. It should be possible in the future to improve this assembly by sequencing large insert libraries that will aid in resolving the repetitive nature and to enlarge scaffolds of the Hva assembly (Raffaele and Kamoun, 2012). An additional approach that might be implemented to improve our current hybrid assembly would be to use a “fosmid-to-fosmid” strategy as that followed by Zheng et al. (2013), who significantly improved an earlier assembly of the *P. striiformis* f.sp. *triticina* genome (Cantu et al., 2011). The GC content of the Hva genome (33%) was lower than *M. larici-populina* (41%), and *P. graminis* f.sp *tritici* (43.3%) (Duplessis et al., 2014). This difference could be explained by GC repetitive sequences collapsing into contigs, therefore yielding a GC content reduction because GC sequences are underrepresented. Though the hybrid Hva genome assembly was highly fragmented, the CEGMA analysis indicated that a significant amount of the genome's gene space was revealed and we consider the current hybrid assembly to be representative of the gene space of a chimeric Hva genome.

Comparative genomics showed considerable similarities between Hva and other rust fungal genomes; over 73% of Hva predicted proteins had homologs among Pucciniales protein datasets. Although rust genomes vary in size, they are very similar in gene content suggesting the presence of a large core set of rust fungus specific genes needed for their pathogenicity. It will also be significant to study the function and specificity of Hva predicted proteins not found in other rusts and study their virulence species-specific adaptations. All in all this set of Hva predicted proteins represent a valuable resource that contributes to the Pucciniales gene repertoire.

In order to capture the gene space of the coffee rust genome, we performed a transcriptome analysis of freshly harvested urediniospores based on Illumina RNA-Seq. The number of secreted proteins predicted in this study is smaller than the number found in *M. larici-populina* (1184 SSPs) and *P. graminis* f.sp. *tritici* (1106 SSPs) genomes (Duplessis et al., 2011), perhaps reflecting the Hva partial genome assembled. Also, it has to be considered that *M. larici-populina* and *P. graminis* f.sp. *tritici* SSPs were predicted with the SignalP, TargetP, and TMHMM algorithms while we did not include TMHMM in our predictions. The non-inclusion of TMHMM transmembrane protein predictions in some way renders our current set of secreted proteins incomplete. On the whole, this set of Hva predicted secreted-proteins is a basic tool for the identification of pathogenicity-related genes as shown for other rusts (Joly et al., 2010; Cantu et al., 2011). It is possible that avirulence elicitors be present among the set of predicted secreted proteins, as it has been found in flax rust (Catanzariti et al., 2006). For Hva proteins KF018008, KF018015, KF018016, KF018020, KF018028 we did not find evidence of predicted homologs in other rusts, representing unique genes of the coffee-rust interaction not represented in other pathosystems; secreted proteins have been found to be lineage-specific in other rusts as well (Duplessis et al., 2011). Overall analysis of the Hva predicted secretome shows that secreted proteins are well conserved among plant rusts and that they include functions most likely involved in the pathogenesis of the fungus. Therefore, this group of annotated secreted proteins suits well as prime candidates for functional testing.

The Hva genome contains most of the gene families coding for signal transduction pathways identified in the genomes of other rust fungi. It is assumed that these gene families are involved in signal perception mechanisms of rust urediniospores (Duplessis et al., 2011) and gives them a highly specialized mechanism for the detection of stomata (Kemen and Jones, 2012). We have grouped the candidate PKs with signal perception roles related to pathogenesis in 18 gene families, those identified in pathogen basidiomycetes but absent in non-pathogenic species.

Illumina and 454 sequencing was used to generate a draft genome in different Hva isolates. Due to the complexity of the genome sequence—similar to other rust fungi- a minimal draft chimeric genome was defined by considering the genome of the different isolates altogether. The genome sequence is a novel resource in Pucciniales, a group that includes many species that are economically major diseases of several crops. It provides data to study the evolution of this important group of plant pathogens. The draft genome sequence of Hva will serve as a template for future assemblies of isolates of this fungus and to understand the molecular mechanisms used by this pathogen to attack the coffee plant and to study its diversity. It will also be the basis for the development of molecular markers to distinguish races/isolates given the enormous difficulties of trying to identify coffee rust races by the use of differential plants. The genomic data of the coffee leaf rust presented here are a reference to track changes in field populations, to characterize the decline in sensitivity against widely used fungicides such as triazoles and strobilurins that are used in coffee rust disease management, and to preserve genotype identity in fungal collections. The increased use of coffee rust-resistant varieties will increase the selective pressure to favor complex fungal genotypes, and resources such as the secretome set is the cornerstone for the development of innovative resistance mechanisms to control this pathogen.

## Materials and methods

### Nuclear DNA content estimated by FCM

Flow cytometry (FCM) was used to estimate nuclear DNA content in urediniospores of *H. vastatrix*, following the protocol described by Eilam et al. (1994), modified for the uredinial stage. Urediniospores were suspended in 0.1% Tween 20 in water for 20 min. The suspension was incubated for 2 min. in a 1000-watt microwave on 50% power level, adding Propidium Iodide and RNase to final concentrations of 4 μg/ml and 50 μg/ml respectively, and incubated for 1 h at 37°C. The urediniospores samples were run on a Bacton Dickinson FACS IV flow cytometer. Urediniospores of *P. triticina* were used as a control. Data from the flow cytometer were analyzed using the Flowing Software v2.5 at the Centre for Biotechnology University of Turku, Finland. Urediniospores of Hva and *P. triticina* were stained and analyzed simultaneously, with the standard control positioned on channel 200. The *C*-Value (pg) was converted to base pairs (bp), considering that 1pg = 978 Mb (Dolezel et al., 2003). *H. vastatrix* samples used for FCM are described in Table 1.

### Genome and transcriptome sequencing and assembly

Hva urediniospore samples were scraped from infected coffee leaves taking care to sample very young pustules with no evidence of the presence of the hyperparasitic fungus *Lecanicillium lecanii*. The coffee genotypes sampled for Hva and the sequencing technologies used are described in Table 2. DNA was extracted using the DNeasy Plant Mini-Kit (Qiagen, Hilden, Germany); *H. vastatrix* DNA samples were used to construct 100 bp paired-end libraries and sequenced by Illumina™ HiSeq 2000 at BGI in China. Single-end libraries were sequenced by ROCHE™ 454 GS FLX Titanium method at Macrogen in Korea.

Reads were subjected to quality control checks using FastQC (Babraham Bioinformatics, Babraham Institute), trimmed using the CLC quality_trim script (CLC bio, Aarhus, Denmark), masked or filtered by low complexity end regions, and exclusion of reads shorter than 70 nucleotides. Mdust and SeqClean were used for the cleaning process (The Gene Index Project, Harvard University—http://sourceforge.net/projects/seqclean/files/). Trimmed and filtered reads were assembled with the CLC Assembly Cell v4.0.1 (CLC bio, Aarhus, Denmark) with the following parameters: deletions penalty = 3, no global alignment, remove duplicates, min contig length = 200 bp, paired-end distance = 200–400 bp. The quality of the assembly was assessed with CLC tools (clc_assembly_viewer, assembly_info) and in-house R scripts available at (http://bioinformatics.cenicafe.org/index.php/wiki/Third_Hybrid_Assembly_of_454_and_Illumina_data_with_CLC).

The hybrid assembly was analyzed using MEGAN 4 (Huson et al., 2001) to assess the level of possible contamination and to perform a first approximation of the biological communities associated with Hva on the coffee leaf. Blastx was performed using the contigs from the hybrid assembly (Illumina + 454 short reads) (396,264 contigs) against the NCBI non-redundant protein database. An *E*-value of 10e^−3^ was used as a cut-off following the recommendation from the MEGAN developers. MEGAN was used to map and visualize the Low Common Ancestor (LCA) in the NCBI tree taxonomy for each contig. With the aim of filtering out putative contaminated sequences, contigs that presented similarities to reported fungal sequences were extracted to form a reliable set of Hva genome contigs. The reliable set of *H. vastatrix* genome contigs was compared against the *P*. *graminis* f.sp. *tritici* and *P. pachyrhizi* mitochondrial genomes using Blastn (with an *E*-value threshold of 1E^−5^).

For RNA-seq sample preparation, Hva urediniospores were scraped from infected coffee leaves of the coffee genotypes described in Table S3, taking care to sample very young pustules with no evidence of the presence of the hyperparasitic fungus *Lecanicillium lecanii*. RNA was extracted from urediniospores using the RNeasy Plant Mini Kit (Qiagen, Valencia, CA, USA). Normalized library construction was performed at Evrogen, Moscow, Russia using Kamchatka crab duplex-specific nuclease (Zhulidov et al., 2004). First-strand cDNA was prepared from poly(A)+ *H. vastatrix* urediniospores RNA using a SMART™ PCR cDNA Synthesis Kit (Clontech), according to the manufacturer's protocol. SMART™ Oligo II and CDS primers (Clontech) were used for first-strand cDNA synthesis. A 1.5 ml aliquot of a 100 ng/ml of the first-strand cDNA solution was incubated for normalization with 0.25 Kunitz units of duplex-specific nuclease from kamchatka crab and amplified by PCR. Sequencing of amplified cDNA products was performed on an Illumina™ HiSeq 2000 system (BGI, Shenzhen, 518083, China).

RNA-seq data were filtered before assembly. The quality of the transcripts was measured using the FASTX-Toolkit, reads were trimmed by quality and duplicates were removed. Clean reads longer than 200bp were assembled using the Trinity package (Grabherr et al., 2011). First, the reads were run through Trinity's Inchworm module, which assembles the read data set into different pools of reads, and the Chrysalis module was used to construct de Brujin graphs for all the read pools obtained using Inchworm. We used the module Butterfly that reconciles de Brujin graphs using the read pools from the former modules and output assembled contigs. We mapped Hva transcript datasets to the HvHybrid 454-Illumina assembly and we also compared transcripts against the NR database to identify plant, bacterial and other contaminant sequences.

### Transposable elements prediction

We surveyed the frequency and classes of TE-like elements present in the HvHybrid assembly using the algorithm RepeatMasker (Smit et al., 1996-2004) and the RepBase12.12 and fngrep.ref databases. The fngrep.ref database included 1726 transposable elements identified in fungi. Novel retrotransposon families were manually annotated from the gene families identified with OrthoMCL (see below).

### Gene prediction

For the prediction of gene models, we followed the “align then assemble” approach (Martin and Wang, 2011). We mapped RNA-seq short reads to the genome using TopHat (Trapnell et al., 2009), and we identified putative transcriptional units using Augustus (Stanke and Waack, 2003). Protein sequences were computationally deduced from the transcriptional units. Gene families from predicted proteins larger than 70 amino acids were identified with OrthoMCL using a default MCL inflation value of 1.5 and a blastp *e*-value of 10e^−5^ (Li et al., 2003). We explored the set of predicted proteins, searching for KOGs using the CD-Search Tool and the Conserved Domain Database (www.ncbi.nlm.nih.gov/Structure/cdd/cdd.shtml).

### Comparative genomics

The Hva genome contigs were aligned against the genomes of *P. graminis*, *M. larici*-*populina* and *U. maydis* using Mauve (Darling et al., 2004). The Low Collinear Block (LCB) values were set through visual inspection by searching the best block size for each pair of alignments (largest coverage of both genomes). Finally, values used for LCB were as follows: *P. graminis* 12,154, *M. larici*-*populina* 10,409, and *U. maydis* 1203.

For genome annotation, we used custom Perl scripts and basic bioinformatics software such as BLAST (Altschul et al., 1990). The databases we used for comparisons corresponded to 67,118 Pucciniales sequences comprising 16,694 protein-coding genes from *M. larici-populina* (Duplessis et al., 2011), 22,815 *P. striiformis* f.sp. *tritici* sequences (Cantu et al., 2011), 15,979 *P. graminis* f.sp. *tritici* sequences (Duplessis et al., 2011), and 11,630 *P. triticina* sequences (Xu et al., 2011).

For homology searches of protein kinases (PKs) we run BLASTp (version 2.2.28) with an *e* = 1e^−10^. We searched *H. vastatrix* predicted proteins against 131 predicted PKs from *Sacharomyces cerevisiae* and then we compared the coffee rust PKs against *M. laricis-populina*, *P. striiformis* f.sp *tritici*, *P. graminis* f.sp *tritici*, *P. triticina*, and *U. maydis* predicted PKs.

### Secreted proteins

The *H. vastatrix* predicted proteins were classified into secreted and non-secreted proteins. For this task, the programs SignalP 4.0 (Petersen et al., 2011) and PProwler (Hawkins and Boden, 2006) were used to predict putatively secreted proteins. A 0.9 probability cut-off was used for PProwler predictions. A set of secreted proteins predicted previously for *H. vastatrix* by Fernandez et al. (2012) was used for comparison with our predictions. Briefly, a Blastp was performed between our set of *H. vastatrix* proteins and the predictions by Fernandez et al. (2012). Finally, a set of proteins that showed similarity (Blastp *e* = 1e^−5^) with the secreted proteins predicted by Fernandez et al. (2012) was obtained. Reciprocal comparisons of the three sets of secreted proteins were performed (SignalP, PProwler and Fernandez-Blastp) to establish the proteins shared by the three predictions.

## Availability

Raw data and metadata for the Genome project is available at NCBI, BioProject ID: PRJNA188788 and the Transcriptome project ID: PRJEB2960. Predicted and secreted proteins are available at http://bioinformatics.cenicafe.org/index.php/wiki/CoffeeRustPredictedProteins.

The hybrid reference assembled genome contigs are available for download at: http://bioinformatics.cenicafe.org/index.php/wiki/CoffeeRustHybridDraftAssembly_Contigs.

### Conflict of interest statement

The authors declare that the research was conducted in the absence of any commercial or financial relationships that could be construed as a potential conflict of interest.
